# *Legionella* Survives and Elongates in Algal Consortia Containing Bacteria in Alkaline Oligotrophic Conditions

**DOI:** 10.1264/jsme2.ME25016

**Published:** 2025-12-03

**Authors:** Wakako Satou, Naohiro Nagai, Masashi Hatamoto

**Affiliations:** 1 Team II, Control & Suppression Technology Department, Basic Technology R&D Group, Innovation Division, Kurita Water Industries Ltd., 1–4–1 Daikanyama, Akishima-shi, Tokyo, Japan; 2 Department of Civil and Environmental Engineering, Nagaoka University of Technology, 1603–1, Kamitomioka-machi, Nagaoka-shi, Niigata-ken, Japan

**Keywords:** *Legionella*, filamentous, alga, bacteria, *in situ* hybridization chain reaction

## Abstract

Cooling towers are a major source of *Legionella*, which causes Legionnaires’ disease. These bacteria grow in predatory organisms; however, the impact of non-predatory organisms in cooling towers on *Legionella* survival and growth remains unclear. Therefore, we investigated the effects of photosynthetic algae, the primary component of biofilms in open cooling water systems, on *Legionella*. We cultivated *Legionella* with algae collected from the towers or with pure algal strain under alkaline conditions and revealed that the *Legionella* 16S rRNA copy number was higher than that of *Legionella* alone. We also exami­ned *Legionella* using an *in situ* hybridization chain reaction and found that some were elongated and exhibited a filamentous morphology on algal cells. Furthermore, *Legionella* was more active when co-cultured with pure algal strain plus *Serratia* spp. than when co-cultured with pure alga alone. 18S rRNA gene sequencing revealed that the algae collected had not previously been reported to coexist with *Legionella*. This result suggests that diverse algae in the environment support the growth of *Legionella*. This is the first study to experimentally demonstrate that algae promote *Legionella* elongation, and also that the coexistence of bacteria furthers this phenomenon. These results provide a new perspective on the ecology of *Legionella* and the role of non-predatory organisms.

Bacteria of the *Legionella* species are ubiquitously distributed in the environment. Cooling water systems are a source of *Legionella* (Fraser *et al.*, 1977; Chamberlain *et al.*, 2017). These bacteria multiply in cooling towers and are released into the environment via the mist exhaust of cooling towers, eventually infecting the human respiratory system and causing legionellosis. Other sources include humidifiers (Mahoney *et al.*, 1992), circulating bathtubs (Joseph *et al.*, 2005), and potting mix or compost (Whiley and Bentham, 2011).

Cooling water is generally oligotrophic and alkaline, which differs from the typical nutrient concentrations and pH of *Legionella* culture conditions. To survive and grow in the conditions present in cooling water, *Legionella* species have been proposed to coexist with or parasitize different types of organisms in biofilms or sediments. Previous studies reported that *Legionella* associated with various organisms in different manners, including the parasitism of predatory microbes, such as amoebae (Grimm *et al.*, 1998; Dietersdorfer *et al.*, 2018) and protozoans (Rasch *et al.*, 2016; Caicedo *et al.*, 2018), and coexistence with non-predatory microbes, such as other bacteria (Taylor *et al.*, 2013), dead bacterial cells (Temmerman *et al.*, 2006), algae (Pope *et al.*, 1982), and photosynthetic microbes (Tison *et al.*, 1980; Bohach and Snyder, 1983a). Many types of microorganisms have been suggested to contribute to the survival and growth of *Legionella*, which is a characteristic feature of *Legionella*. Amoebae are known to function as a *Legionella* host (Rowbotham, 1980). Amoebae feed on non-predatory organisms, such as algae and bacteria forming biofilms in cooling tower. During this process, they ingest *Legionella*, which survive and multiply within amoeba cells and are eventually released in cooling systems (Anand *et al.*, 1983; Oliva *et al.*, 2018). The parasitism of amoebae by *Legionella* has been extensively exami­ned. The mechanism by which *Legionella* multiplies in the vacuoles of amoebae was described by Vogel *et al.* (1998). Many studies have reported the proliferation of *Legionella* in amoebae, which have been detected in most cooling water systems (Zeybek *et al.*, 2017). Collectively, these findings suggest that *Legionella* multiplication in cooling towers is attributed to the presence of amoebae. However, *Legionella* are not always detected in water systems occupied by amoebae and, conversely, the presence of *Legionella* does not guarantee the coexistence of amoebae (Scheikl *et al.*, 2016). Furthermore, the proliferation of *Legionella* in protozoa other than amoebae has demonstrated (Barbaree *et al.*, 1986). Barbaree *et al.*, (1986) reported cooling water systems in which the protozoa isolated did not exhibit *Legionella* proliferation as well as systems in which *Legionella* were detected, but protozoa were not isolated. These findings indicate that there are still some aspects to be investigated regarding whether protozoa are the sole site for *Legionella* proliferation in cooling water systems. Therefore, we hypothesized that organisms other than predatory protozoa coexisting with *Legionella*, particularly non-predatory organisms that are dominant in systems in which *Legionella* have been detected, may contribute to the proliferation of *Legionella* in this system.

To examine organisms other than protozoa that enable *Legionella* multiplication in cooling water systems, we selected algae as a candidate photosynthetic microbe because 1) photosynthetic microbes become dominant in open cooling systems (Di Gregorio *et al.*, 2017) and 2) algae contribute to *Legionella* proliferation. However, cocultures of *Legionella* with algae are typically performed at pH 7.5 (Pope *et al.*, 1982). It remains unclear whether algae contribute to the growth and survival of *Legionella* at the pH that generally prevails in cooling water systems (pH 8–9). In addition, we investigated whether bacteria contributed to the survival of *Legionella* because algae and bacteria commonly coexist in natural environments. Previous studies showed that bacteria isolated from environments supported the multiplication of *Legionella*. For example, *Flavobacterium breve* isolated from a hot water tank (Wadowsy and Yee, 1983) or *Pseudomonas vesicularis* (Koide *et al.*, 1989) from a shower hose induced the satellite growth of *Legionella* around their colonies on an agar medium that did not support *Legionella* growth alone. Furthermore, *Legionella* were shown to persist within *Pseudomonas fluorescens* biofilms while maintaining its colony-forming ability (Stewart *et al.*, 2012; Silva *et al.*, 2024). However, it has yet to be demonstrated that bacteria from cooling water systems support the persistence of *Legionella*. Additionally, the effects of bacteria coexisting with algae on *Legionella* growth or survival have not yet been exami­ned.

In planning this study, we considered it important to conduct morphological observations to clarify the relationships between symbiotic organisms and *Legionella*. The coexistence of *Legionella* with algae is typically observed using optical microscopy (Bohach and Snyder, 1983b). However, fluorescence *in situ* hybridization (FISH) has recently been used to detect *Legionella* coexisting with microbes in the environment (Taylor *et al.*, 2013; Rasch *et al.*, 2016; Caicedo *et al.*, 2018). Since many studies have used the LEG705 probe as a *Legionella* genus–specific FISH probe (Manz *et al.*, 1995), we initially applied this probe to an algal and bacterial consortium containing *Legionella*.

We herein investigated the effects of algae on *Legionella* growth in an alkaline oligotrophic environment and reported the results obtained.

## Materials and Methods

### Microbial strains, cultures, and identification methods

*Legionella pneumophila* (ATCC33152) was cultured using buffered charcoal yeast extract (BCYEα) *Legionella* agar medium (OXOID). *Vischeria stellata* NIES-2148, an axenic algal strain, was purchased from the Microbial Culture Collection at the National Institute for Environmental Studies. All algal strains were grown on C medium (Microbial Culture Collection, National Institute for Environmental Studies. URL https://mcc.nies.go.jp/02medium.html#c). *Serratia marcescens* was isolated from biofilm samples collected from cooling water systems by single-colony isolation using R2A agar plates. The 16S rRNA gene sequence was elucidated at FASMAC using a MicroSEQ^TM^ Full Gene 16S rDNA PCR Kit with a MicroSEQ ID system (Thermo Fisher Scientific). *S. marcescens* was subsequently grown on R2A agar.

Biofilms were collected from two different cooling towers where *Legionella* had previously been detected. Specifically, biofilms were collected using a 1-mL or 10-mL syringe from the area in the tower with green biofilm growth, which was either the pit wall or the filler (Fig. S1 and S2). Each biofilm was then diluted using sterile tap water and subsequently cultured in C medium. A type of algae colony was isolated from each cooling tower, which resulted in two types of algae ultimately being isolated. The identification of the isolated algal strains by sequencing the 18S rRNA gene was performed at Techno Suruga Laboratory. Briefly, total genomic DNA was extracted using the Nucleo Spin Plant II Kit (MACHEREY-NAGEL), and was then used for PCR amplification of the 18S rRNA gene using the primers SR-1 (5′-TAC CTG GTT GAT CCT GCC AG-3′) and SR-12 (5′-CCT TCC GCA GGT TCA CCT AC-3′) (Nakayama *et al.*, 1996). Sequencing was performed using the BigDye Terminator V3.1 Cycle Sequencing Kit (Thermo Fisher Scientific) on an ABI PRISM 3500xl Genetic Analyzer (Thermo Fisher Scientific).

### Coculture tests for *L. pneumophila* and algae

The two types of algae isolated from the cooling towers were used. They were named the *V. stellata* consortium and *Klebsormidium elegans* consortium. In coculture tests, 50‍ ‍mL of MA medium (pH 8.6) (Microbial Culture Collection, National Institute for Environmental Studies. URL https://mcc.nies.go.jp/02medium.html#ma) was prepared in a 100-mL conical flask and then inoculated with *L. pneumophila* along with the *V. stellata* or *K. elegans* consortia. Inoculated organisms were prepared as follows: *L. pneumophila* grown on BYCEα agar was diluted with sterile tap water to attain an absorbance of 0.1 at 662‍ ‍nm, the estimated *Legionella* CFU was approximately 10^6^–10^7^ CFU μL^–1^. This solution was added to MA medium at a diluted rate of 1/1,000 (Table 1) or 1/100,000 (Table 2). The *V. stellata* and *K. elegans* consortia grown on C medium were collected by centrifugation and washed with MA medium. One milliliter of the *V. stellata* consortium (OD_662_, 0.1) or 3‍ ‍mL of the *K. elegans* consortium (OD_662_, 0.03) was added to the MA medium. Due to the lack of previous studies on the cultivation of algae, cocultures were performed under various conditions: in a growth chamber at 36°C with fluorescent light (photon flux density: 105–110‍ ‍μmol m^–2^ S^–1^), at 26 or‍ ‍34°C with LED light (photon flux density: 2–18‍ ‍μmol m^–2^ S^–1^), or at room temperature (typically 26–28°C; minimum, 24°C; maximum, 29°C) under natural sunlight (light flux density: 8‍ ‍μmol‍ ‍m^–2^‍ ‍S^–1^) from a north-facing window without additional light. All experiments were conducted under static conditions. We conducted tests in one series because we were unable to obtain a sufficient amount of the algae used in our experiments due to the lack of previous cultivation reports.

The culture medium was sampled over time, and *Legionella* CFUs, the copy numbers of the 16S rRNA gene and 16S rRNA, and the CFUs of heterotrophic bacteria were measured. In addition, algal growth was visually observed, and the chlo­rophyll concentration per unit dry weight was quantified.

### *Legionella* quantification

#### Measurement of *Legionella* CFUs

*Legionella* CFUs were quantified using glycine, vancomycin, polymyxin B, and cycloheximide (GVPC) *Legionella* agar medium. The GVPC agar medium was prepared using *Legionella* agar base (OXOID), *Legionella* BCYE growth supplement (OXOID), and *Legionella* (GVPC) selective supplement (OXOID) according to the manufacturer’s instructions. The collected culture medium was diluted with sterilized tap water; 100-μL aliquots were mixed with the same amount of KCl–HCl buffer (pH 2.2; KANTO Chemical) and plated on GVPC agar plates. The plates were incubated at 36°C for 7–10 days, the number of grey–white colonies was counted, and 5–10 were then selected for the identification of *Legionella*. Colonies that did not grow on BCYEα-cysteine agar (OXOID), but grew on BCYEα agar medium were identified as cysteine-requiring colonies, which were confirmed through agglutination using *Legionella pneumophila* sero1 serum (Denka Seiken). Agglutinated colonies were identified as *L. pneumophila* sero1. The detection limit was 10^–2^ CFU μL^–1^.

#### DNA/RNA extraction

Approximately 100‍ ‍μL of the sample was added to a 2-mL screw-cap tube containing 1‍ ‍g of zirconia beads with a diameter of 0.1‍ ‍mm and 1‍ ‍mL of extraction buffer (100‍ ‍mM Tris-HCl [pH 8.0], 100‍ ‍mM EDTA [pH 8.0], 100‍ ‍mM phosphate buffer [pH 8.0], and 1.5 M NaCl). Screw-cap tubes were loaded into a bead-beating homogenizer (Fisherbrand Bead Mill24), and bead beating was performed for 2‍ ‍min. Thereafter, 110‍ ‍μL of 20% sodium dodecyl sulfate (SDS) was added and the mixture was vortexed vigorously for 30‍ ‍s and incubated at 60°C for 30‍ ‍min. Bead beating was performed again for 2‍ ‍min before centrifugation at 12,000×*g* at 24°C for 15‍ ‍min. Seven hundred microliters of the supernatant was transferred to a 1.5-mL tube, and the same amount of chloroform: isoamyl alcohol (24:1) was added, followed by mixing and centrifugation at 7,500×*g* at 24°C for 5‍ ‍min. A total of 630‍ ‍μL of the supernatant was then transferred to a fresh 1.5-mL tube for isopropanol DNA precipitation. Precipitated DNA was dissolved in TE buffer and purified using a MonoFas DNA Purification Kit I (ANIMOS) according to the manufacturer’s instructions. This DNA was subjected to PCR for the quantification of the *Legionella* 16S rRNA gene copy number.

Approximately 100‍ ‍μL of the sample was added to a 2-mL screw-cap tube containing 1‍ ‍g of zirconia beads with a diameter of 0.1‍ ‍mm, and 1‍ ‍mL of ISOGEN (NIPPON GENE) was added. RNA extraction was conducted according to the manufacturer’s protocol. Total extracted RNA was used for cDNA synthesis using the PrimeScript^TM^ II 1st strand cDNA Synthesis Kit (Takara Bio). This cDNA was subjected to PCR for the quantification of the *Legionella* 16S rRNA copy number.

### Quantitative PCR

The hybridization probe method was performed for qPCR. The LEG225–LEG858 primer pair (Miyamoto *et al.*, 1997) was used for amplification with the detection probes Leg_16S1-S (5′-AGT GGC GAA GGC GGC TAG CT-3′) with a FITC label at the 3' end and Leg_16S2-S (5′-TAC TGA CAC TGA GGC ACG AAA GCG T-3′) with an LCRed640 label at the 5' end. TaKaRa Ex Taq® Hot Start Version (Takara Bio) was used for PCR amplification. Total DNA extracted from *L. pneumophila* grown on BYCEα agar medium was used to construct a standard curve. Light Cycler II (Roche) was used for thermal cycling under the following conditions: initial denaturation at 95°C for 10 s; 50 cycles of denaturation at 95°C for 10‍ ‍s, annealing at 57°C for 15‍ ‍s, and extension at 72°C for 30 s; final cooling at 40°C for 5 s. qPCR was performed in duplicate. The detection limit of DNA or RNA was 1 copy μL^–1^.

### Quantification of heterotrophic bacteria grown on R2A agar plates

Heterotrophic bacteria were counted using R2A agar plates (Gibco). The collected culture medium was diluted with sterilized tap water and inoculated onto the plates. The plates were then incubated at 30°C for 2–7 days, and the number of colonies was counted. The detection limit was 10^–2^ CFU μL^–1^.

### Chlorophyll extraction and anal­ysis

One milliliter of the culture was collected, and the medium was removed by centrifugation. Ice-cool acetone (800‍ ‍μL) was added, and chlo­rophyll was extracted from the collected samples by incubating for 15‍ ‍min in the dark with occasional mixing. After centrifugation, the supernatant was collected, the liquid volume was measured, and 2.5‍ ‍mM sodium phosphate buffer (pH 7.8) was added to prepare an 80% acetone solution. Absorbance at 750, 663.6, and 646.6‍ ‍nm was measured using a spectrophotometer; absorbance at 750‍ ‍nm was used as the background and was subtracted from absorbance at 663.6 and 646.6‍ ‍nm. These values were converted to chlo­rophyll concentration per unit volume using the formula of Porra *et al.* (1989):

chlo­rophyll a (μg mL^–1^)=12.25×A_663.6_–2.55×A_646.6_.

### Dry weight measurements of algal consortia

A glass filter (Whatman) with a pore size of 1‍ ‍μm and diameter of 47‍ ‍mm was thoroughly washed with ultrapure water, dried at 105°C, returned to room temperature in a desiccator, and weighed. One milliliter of the culture solution was filtered through this filter, which was then incubated at 105°C for 2 h, returned to a room temperature in a desiccator, and weighed. The original weight of the glass filter was subtracted from the weight after filtration to yield the dry weight in mg. Subsequently, chlorophyll concentration per unit volume was converted on a dry weight basis.

### Sample fixation for FISH and *in situ* Hybridization Chain Reaction (*in situ* HCR)

Pure cultures of *L. pneumophila* or mixed cultures with algal consortia or pure algae were harvested by centrifugation. Samples were washed twice with phosphate-buffered saline (PBS) by centrifugation and then fixed with 4% paraformaldehyde in PBS at room temperature (24–26°C) for 30‍ ‍min. After fixation, samples were washed twice with PBS and stored in 50% ethanol with PBS at –20°C.

### FISH

To detect *L. pneumophila*, the 16S rRNA–targeting oligonucleotide probe LEG705 (5′-CTG GTG TTC CTT CCG ATC-3′), which targets sequences from the *Legionellae* family (Manz *et al.*, 1995), was used. The EUB338 probe (5′-GCT GCC TCC CGT AGG AGT-3′), complementary to a region of 16S rRNA conserved in the domain Bacteria, was used as the positive control for FISH, and the non-EUB338 probe (5′-ACT CCT ACG GGA GGC AGC-3′), complementary to the EUB338 probe, was used to detect non-specific binding (Amann *et al.*, 1990). The probes used were labeled with Alexa488 at the 5' end. Hybridization was performed at 60°C for 1.5‍ ‍h or overnight in hybridization buffer (0.9 M NaCl, 20‍ ‍mM Tris-HCl [pH 7.2], 0.01% SDS, and 0% formamide) containing 5‍ ‍ng μL^–1^ of a labeled probe. To remove unhybridized probes, the glass slide was gently rinsed (0.18 M NaCl, 20‍ ‍mM Tris-HCl [pH 7.2], and 0.01% SDS) and immersed in wash buffer at 46°C for 15‍ ‍min. The slides were carefully rinsed with ultrapure water and air-dried; if necessary, one drop of ProLong Gold Antifade Reagent with DAPI (Thermo Fisher Scientific) was applied to each well. Cells were visualized using an epifluorescence microscope (BX52; Olympus) or a confocal laser scanning microscope (CLSM) (Zeiss LSM 780; Zeiss). Approximately 10 or more fields of view were observed for each sample.

### *In situ* HCR

*In situ* DNA-HCR was based on the procedure described by Yamaguchi *et al.* (2015) with some modifications. We used LEG705, EUB338, and non-EUB338 probes, which have an initiating sequence, and the amplifying probes H1 and H2. Supplementary Table S1 lists probe sequences. Fixed cells were mounted in all wells of a 12- or 24-well glass slides. Samples were dehydrated using an ethanol series (50, 80, and 96% [v/v] ethanol for 3, 1, and 1‍ ‍min, respectively). Hybridization buffer (20‍ ‍mM Tris-HCl [pH 7.5], 0.9 M NaCl, 0.01% SDS, 10% dextran sulfate, 1% blocking reagent, and 35% formamide) containing initiator probes (0.5‍ ‍μM) was added at 10‍ ‍μL per well. Glass slides were placed in a humidified chamber and incubated at 46°C overnight. To remove the excess probe, the glass slide was gently rinsed and immersed in wash buffer (0.07 M NaCl, 20‍ ‍mM Tris-HCl [pH 7.2], and 0.01% SDS) at 48°C for 30‍ ‍min. The H1 and H2 amplification probes were incubated separately at 5‍ ‍μM each in amplification buffer (50‍ ‍mM Na_2_HPO_4_, 0.9 M NaCl, 0.01% SDS, 10% dextran sulfate, and 1% blocking reagent) at 95°C for 1.5‍ ‍min and then at 25°C for 30‍ ‍min; the buffers containing the two probes were then mixed. Ten microliters of the H1 and H2 amplification probe mixture was added to each well and the samples were incubated at 35°C for 1‍ ‍h in a humidified chamber. To remove excess amplifier probes, slides were then immersed in 50‍ ‍mL of 1× PBS containing 0.01% SDS at 4°C for 10‍ ‍min. Slides were immersed in ultrapure water and 96% ethanol at room temperature for 30‍ ‍s each and then stored at 4°C. If necessary, one drop of ProLong Gold Antifade Reagent with DAPI (Thermo Fisher Scientific) was added to each well. Cells were visualized using an epifluorescence microscope (BX52; Olympus) or CLSM (Zeiss LSM 780; Zeiss). Approximately 10 or more fields of view were observed for each sample. In the present study, filamentous *Legionella* were operationally defined as cells measuring 20 μm or longer. However, when cocultured with algal cell clusters, the complete visualization of *Legionella* cells was occasionally impeded. Under these conditions, cells measuring between 10–20 μm were also classified as filamentous.

## Results

### Cocultivation of *Legionella* with algal consortia

Microscopic observations of the biofilms collected from the cooling towers revealed a single dominant algal species in each cooling tower. To isolate these algae, samples were serially diluted and cultured on C medium. Two distinct algal species were isolated, each originating from the biofilms of two separate cooling towers. A BLAST search was performed on 1,700 base pairs of the 18S rRNA gene sequences of isolated algae, which identified as *V. stellata* and *K. elegans* with homology scores of 100%. *V. stellata* belongs to *Eustigmatophyceae* and *K. elegans* to the algal genus *Klebsormidium*. Although the algae were isolated, they were in a consortium with bacteria. Therefore, they were named the *V. stellata* and *K. elegans* consortia, and we used an algal isolate containing bacteria for cocultivation with *Legionella*.

Culture conditions and the results obtained are shown in Table 1. All cultures, including the coculture of *Legionella* with algae or *Legionella* alone, were conducted in an oligotrophic environment using the MA medium, which does not contain L-cysteine. The pH of the MA medium was initially set at 8.6 and was maintained at pH 8.0–8.6 for the duration of the culture. Since one of the cooling towers used to obtain samples for the present study is well-maintained and cleaned monthly, the culture duration was set to approximately 1 month and was extended if necessary. An LED was used as the light source in cultures of *Legionella* alone (Table 1 No. 3), and light intensity was set to less than sunlight intensity (Table 1 No. 1 and 2).

**Fig. 1.**
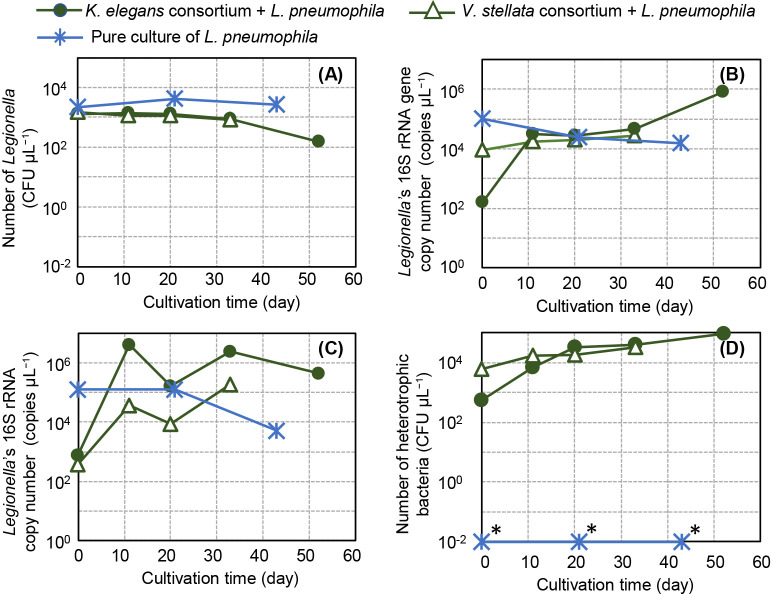
Cultivation of *Legionella pneumophila* with the *Vischeria stellata* or *Klebsormidium elegans* consortium at room temperature (26–28°C) under natural sunlight. (A) Count of *Legionella* CFUs. (B) Copy number of the 16S rRNA gene of *Legionella*. (C) Copy number of the 16S rRNA of *Legionella*. (D) Count of heterotrophic bacteria grown on R2A agar plates. The cultivation of pure cultures of *L. pneumophila* is shown as a control. Detailed culture conditions are shown in Table 1. Each point represents a single measurement. The detection limit of DNA or RNA was 1 copy μL^–1^, while that of *Legionella* and heterotrophic bacteria was 10^–2^ CFU μL^–1^. Data points marked with an asterisk (*) indicate values below the detection limit.

Changes in *Legionella* numbers in the culture were monitored by counting CFUs and measuring the copy numbers of the 16S rRNA gene and 16S rRNA. The number of *Legionella* cells was evaluated by quantifying the 16S rRNA gene copy number, while their activity was assessed by quantifying the 16S rRNA copy number. The results obtained at 26–28°C are shown in Fig. 1A, B, C, and D. Although the MA medium for algae is an oligotrophic medium that does not contain L-cysteine, *Legionella* CFUs were maintained for approximately 1 month in cultures of *Legionella* alone or in those with algal consortia (Fig. 1A). At 0 h, the copy numbers of the 16S rRNA gene and 16S rRNA of *Legionella* cocultured with algal consortia were 10- to 100-fold lower than those in the pure culture of *Legionella*. However, CFU counts were similar between the coculture and pure culture. (Fig. 1A, B, and C). The reason for this difference was unclear; therefore, we focused on changes over time excluding 0 h. During one month of the culture, the 16S rRNA gene copy number of *Legionella* cocultured with algal consortia was sustained at those levels, whereas that of *Legionella* cultured alone slightly decreased (Fig. 1B). Furthermore, differences were observed in the 16S rRNA copy number between *Legionella* cultured alone and those cocultured with algal consortia (Fig. 1C). These results suggest that the algal consortia exerted a positive effect on maintaining the viability or activity of *Legionella.* Fig. 1D shows that the CFU count of heterotrophic bacteria on R2A agar plates changed over time. During the culture period, heterotrophic bacteria multiplied in the coculture with algal consortia, and the CFUs of heterotrophic bacteria were 10- to 100-fold higher than *Legionella* CFUs on R2A agar plates. CFUs were not detected in the culture of *Legionella* alone, indicating no bacterial contamination.

**Fig. 2.**
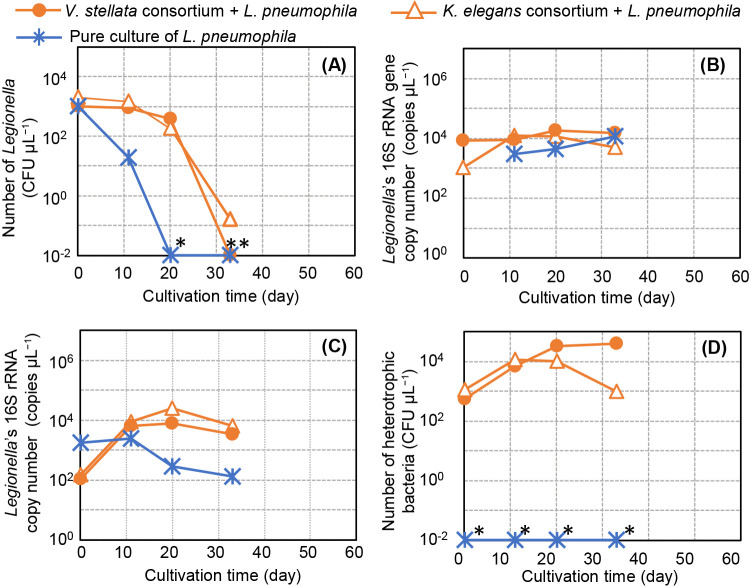
Cultivation of *Legionella pneumophila* with the *Vischeria stellata* or *Klebsormidium elegans* consortium at 36°C under a fluorescent lamp. (A) Count of *Legionella* CFUs. (B) Copy number of the 16S rRNA gene of *Legionella*. (C) Copy number of the 16S rRNA of *Legionella*. (D) Count of heterotrophic bacteria grown on R2A agar plates. The cultivation of pure cultures of *L. pneumophila* is shown as a control. Detailed culture conditions are shown in Table 1. Each point represents a single measurement. The detection limit of DNA or RNA was 1 copy μL^–1^, while that of *Legionella* and heterotrophic bacteria was 10^–2^ CFU μL^–1^. Data points marked with an asterisk (*) indicate values below the detection limit.

A coculture was then performed at 36°C, the optimum culture temperature for *Legionella*. Table 1 No. 4, 5, and 6 show culture conditions and algal growth. Under these conditions, a marked increase was not observed in algal growth and the algal cell color turned white, which were attributed to algal death. Fig. 2A shows the change in *Legionella* CFUs over time. Unlike the results shown in Fig. 1A, *Legionella* CFUs decreased in cocultures and in cultures of *Legionella* alone. The fastest decrease in *Legionella* CFUs was observed in cultures of *Legionella* alone, with CFUs not being detectable after 20 days. In cocultures with algal consortia, the CFU decrease was suppressed; however, after more than 20 days, an immediate reduction was noted. Fig. 2B shows changes in the 16S rRNA gene copy number over time. Although CFUs were not detectable (Fig. 2A), the 16S rRNA gene copy number did not markedly change under any culture condition. Fig. 2C shows changes in the *Legionella* 16S rRNA copy number over time. The 16S rRNA copy number of *Legionella* cultured alone slightly decreased, whereas that *Legionella* cocultured with algal consortia did not. The number of colonies on the R2A agar plate indicated that the algal consortia contained a high concentration of bacteria (Fig. 2D). However, CFU increases stopped after day 20 of the culture, which was attributed to algal death. According to these results, algae did not grow and *Legionella* CFUs decreased under all conditions, which was different from the results shown in Fig. 1. This may have been due to differences in the culture temperature or light source conditions.

### *In situ* HCR of the *Legionella* morphology in algal consortia

**Fig. 3.**
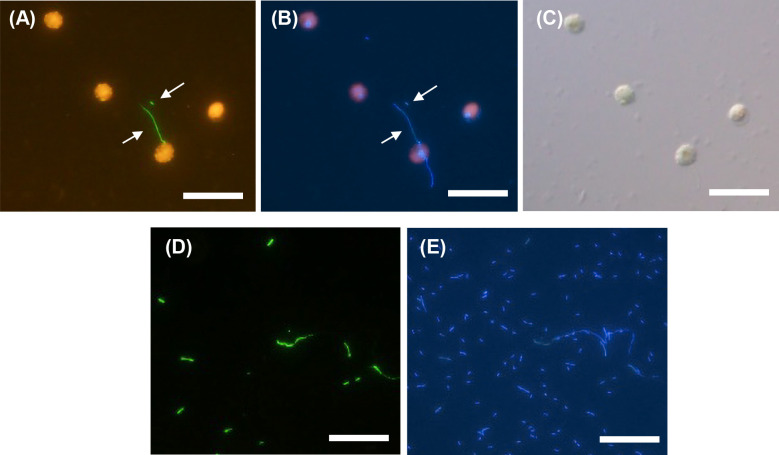
*In situ* HCR detection of *Legionella pneumophila* by the LEG705 initiator probe with Alexa488-labeled H1 and H2 amplifier probes (A), DAPI staining (B), and bright-phase imaging (C) for *L. pneumophila* with the *Vischeria stellata* consortium after 27 days of culture (Culture No. 1 in Table 1). *In situ* HCR detection of *L. pneumophila* by the LEG705 initiator probe with Alexa488-labeled H1 and H2 amplifier probes (D) and DAPI staining (E) for *L. pneumophila* cultured alone on day 42. Scale bars represent 20‍ ‍μm. Ten or more fields of view were observed for each sample.

To detect *Legionella* cells in the coculture with the *V. stellata* consortium (Table 1 No. 1), we initially performed conventional FISH (Supplementary Fig. S3). We observed the presence of DAPI-stained rod-like bacteria in the proximity of algae. Weak green fluorescence was detected (Fig. S3A); however, it was not possible to assess their morphology. This likely corresponded to the *Legionella*-targeted LEG705 probe labeled with the fluorescent dye Alexa488. Since it was challenging to detect *Legionella* associated with algae using conventional FISH, we performed *in situ* HCR, which resulted in clear fluorescence signals from the LEG705 probe that enabled *Legionella* detection. *Legionella* cells appeared as filaments extending from the algal surface and also exhibited a rod-shaped morphology (Fig. 3A and B). In contrast, *Legionella* cells that were cultured alone for 42 days (Table 1 No. 3) and stained with *in situ* HCR showed green fluorescence in the form of rods, whereas no filament-shaped cells were found (Fig. 3D). Moreover, a higher number of rods were stained with DAPI than with green fluorescence (Fig. 3E). The same sample was subjected to *in situ* HCR on day 34, and green fluorescence derived from LEG705 was observed even among algal aggregates (Fig. S4).

**Fig. 4.**
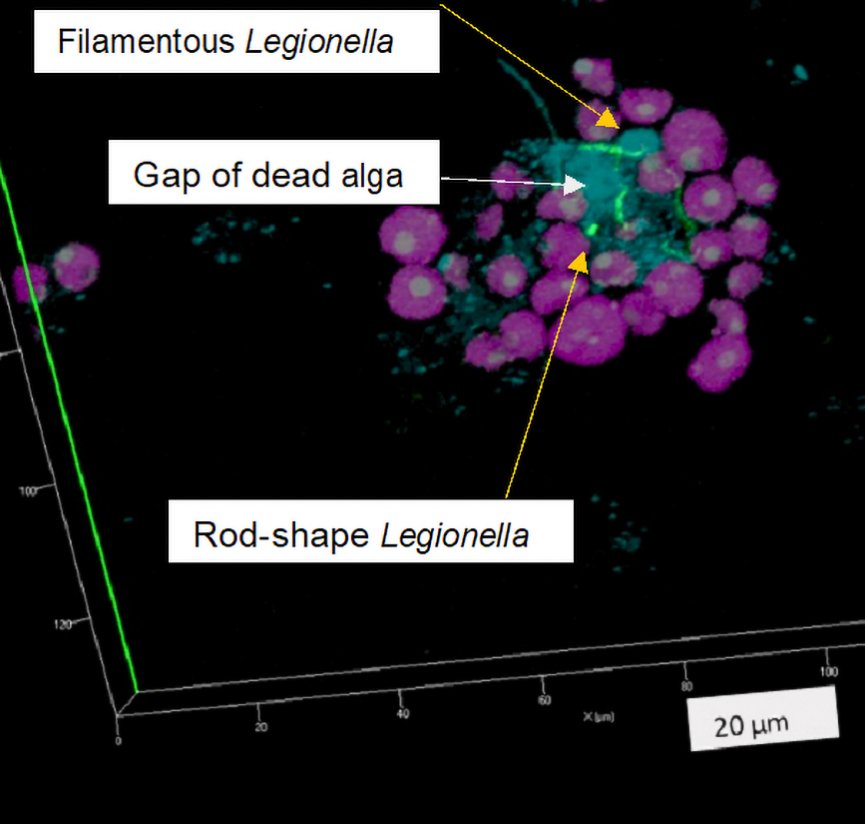
Confocal laser scanning microscopy image of *in situ* HCR detection of *Legionella* in the *Vischeria stellata* consortium. The bright green signal was from *Legionella* cells detected using the *Legionella*-specific LEG705 initiator probe with Alexa488-labeled H1 and H2 amplifier probes. Reddish purple indicates algae, and the blue signal is from DAPI staining. The scale bar represents 20‍ ‍μm. Ten or more fields of view were observed for each sample.

Cells were treated with LEG705 and stained with DAPI, and autofluorescence was imaged using CLSM (Fig. 4). Cells that reacted with the LEG705 probe were considered to be *Legionella*, those that stained with only DAPI were regarded as other bacteria, and autofluorescent cells were considered to be algae. Many bacilli and cocci were detected in the spaces between algal cells, and among them, filamentous or rod-shaped *Legionella* was noted. Filamentous *Legionella* cells extended along the cell surfaces of multiple algae (with gaps present within filamentous *Legionella*). Fig. 1A and D show that there were 100- to 1,000-fold more bacterial CFUs than *Legionella* CFUs, which was supported by the results of staining experiments. Therefore, the voids among algal cells appeared to be filled with bacteria, forming bacterial microcolonies, and *Legionella* cells were also elongated in microcolonies. These results demonstrate that algae, *Legionella,* and other bacteria coexisted in the consortium.

**Fig. 5.**
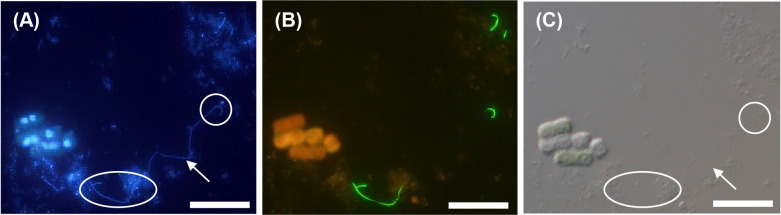
*In situ* HCR observation of *Legionella* cocultured with the *Klebsormidium elegans* consortium. Green fluorescence indicates *Legionella* detected by the LEG705 initiator probe with the Alexa488-labeled H1 and H2 amplifier probes, and orange fluorescence indicates algal autofluorescence (B), DAPI staining (A), and bright-phase imaging (C). Arrows indicate filament-like morphologies. The circle indicates the overlapping area with fluorescence from the LEG705 probe. The scale bar represents 20‍ ‍μm. Ten or more fields of view were observed for each sample.

On day 27, samples cocultured with the *K. elegans* consortium at room temperature (Table 1 No. 2) showed long filamentous bacteria stained with DAPI, and the longest bacterium size was estimated to be 50–100‍ ‍μm (Fig. 5A). Green fluorescence from the LEG705 probe appeared as short filaments (Fig. 5B). This fluorescence overlapped with the ends of the filamentous bacteria stained with DAPI, which indicated that the long filaments were *Legionella*. The detection of green fluorescence from the LEG705 probe suggested a concentrated presence of *Legionella* 16S rRNA, implying that both ends of filamentous bacteria were active.

### Coculture of *Legionella* with pure *V. stellata* NIES2148

The algae isolated in the present study contained several types of bacteria; therefore, an unknown factor from coexisting bacteria may have affected the development and morphology of *Legionella*. We prepared a pure coculture with *V. stellata* strain NIES2148 and *L. pneumophila* to observe the morphology of *Legionella*. All *in situ* HCR images of *Legionella* and algae before the coculture experiment are shown in Fig. S5A, B, and C. Coculture conditions are shown in Table 2, Culture No. 7, 8, and 9.

**Fig. 6.**
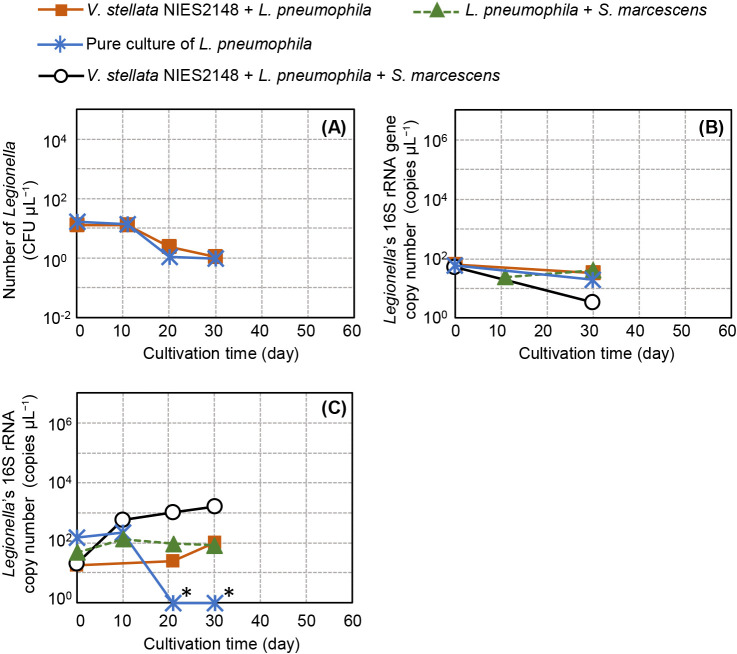
Culture of *Legionella pneumophila* with pure *Vischeria stellata* NIES2148 and *Serratia marcescens*. (A) Number of *Legionella* CFUs. (B) Copy number of the 16S rRNA gene of *Legionella*. (C) Copy number of the 16S rRNA of *Legionella*. The incubation temperature was 34°C. Detailed cultivation conditions are shown in Table 2. Each point represents a single measurement. The detection limit of DNA or RNA was 1 copy μL^–1^, while that of *Legionella* was 10^–2^ CFU μ^–1^. Data points marked with an asterisk (*) indicate values below the detection limit.

In the present study, a fluorescent lamp was used as the light source, and light intensity was less than 20‍ ‍μmol‍ ‍m^–2^‍ ‍s^–1^. The medium containing *V. stellata* NIES2148 turned green and algal growth was observed. Fig. 6 shows changes in the *Legionella* CFU count and the copy numbers of the 16S rRNA gene and 16S rRNA over time. The *Legionella* CFU count and 16S rRNA gene copy number both slightly decreased, which was similar for *Legionella* cultured alone and in combination with *V. stellata* NIES2148 (Fig. 6A and B). However, changes in the *Legionella* 16S rRNA copy number differed between the *Legionella* pure culture and coculture. It decreased to undetectable levels by day 20 in the *Legionella* alone culture, but was maintained and then increased by day 30 in the coculture with algae (Fig. 6C).

**Fig. 7.**
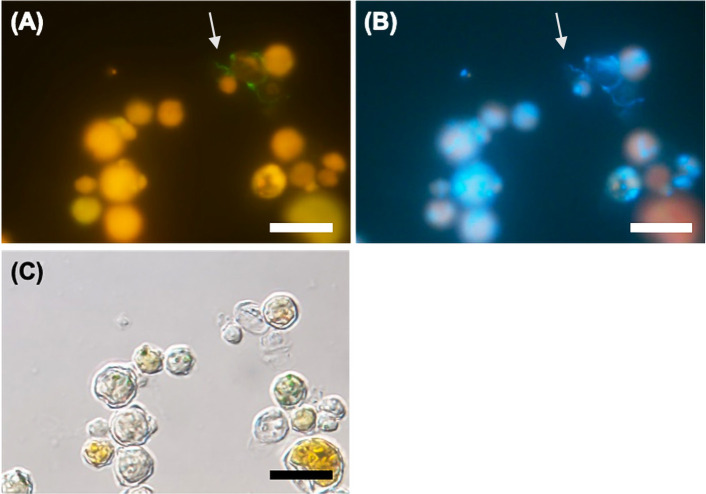
Coculture of *Legionella pneumophila* with pure *Vischeria stellata*. *L. pneumophila* was detected by *in situ* HCR using the LEG705 initiator probe with Alexa488-labeled H1 and H2 amplifier probes (A), DAPI staining (B), and bright-phase imaging (C). Arrows indicate filamentous morphologies of *L. pneumophila*. Scale bars represent 20‍ ‍μm. Ten or more fields of view were observed for each sample.

After *V. stellata* and *Legionella* were cocultured for 30 days, *in situ* HCR showed faint green filamentous fluorescence derived from LEG705 along the algal surface (Fig. 7A), and a DAPI-stained filamentous form corresponding to green fluorescence was observed (Fig. 7B). Additionally, since algae cells appeared to be hollowed out, they were regarded as dead cells (Fig. 7C). On the other hand, in the culture with *Legionella* alone, green fluorescence was not observed, and only rod-shaped *Legionella* cells were detected by DAPI staining.

Observations of cocultures of *Legionella* and the *V. stellata* consortium showed the coexistence of algae, *Legionella*, and other bacteria, and also the elongation of *Legionella* cells in this culture (Fig. 4). To clarify whether the other bacteria contributed to the development of *Legionella*, a coculture of *Legionella*, algae, and other bacteria was performed. Experimental conditions are shown in Table 2, Culture No. 10 and 11. *S. marcescens* isolated from a cooling system was used as the other bacterium. The LEG705 probe did not react with *S. marcescens* (Fig. S5D and E). *Legionella* CFUs were not measured because after the addition of *S. marcescens*, gray white colonies had increased by more than 100-fold on the GVPC agar plate, but were not identified as *Legionella*, while the number of *S. marcescens* CFUs on the R2A agar plate was 100-fold higher than that of *Legionella* CFU. Fig. 6B shows that the *Legionella* 16S rRNA gene copy number changed over time, and decreased more in the triculture system than in the culture of *Legionella* alone or in the coculture with *V. stellata* NEIS2148 or *S. marcescens*. The reason for this difference was unclear. The 16S rRNA copy number in the coculture was maintained, whereas that in the triculture showed an increase by an order of magnitude (Fig. 6C). Additionally, the 16S rRNA copy number in the coculture of *S. marcescens* and *Legionella* did not decrease and was maintained at the same level as that in the algal cocultures.

**Fig. 8.**
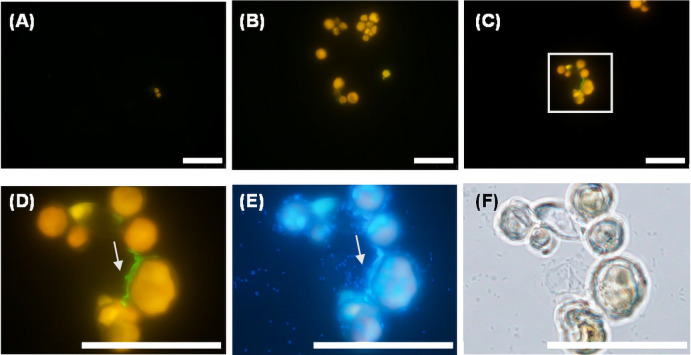
Pure tri-culture of *Vischeria stellata*, *Serratia marcescens*, and *Legionella pneumophila* on days 1 (A), 10 (B), and 21 (C, D, E, F). *L. pneumophila* was detected by *in situ* HCR using the LEG705 initiator probe with Alexa488-labeled H1 and H2 amplifier probes (A, B, C, D), DAPI staining (E), bright-phase imaging (F). Panels D, E, and F are enlarged images of the square area in panel C. Scale bars represent 60‍ ‍μm. Ten or more fields of view were observed for each sample.

Fig. 8 shows *in situ* HCR results for the pure triculture of *V. stellata*, *S. marcescens*, and *L. pneumophila*. Although filamentous green fluorescence derived from the LEG705 probe was not observed until day 10, it was detected on the surface of the algae on day 21 of the culture, which was considered to indicate the presence of *Legionella*. These images were taken under the same conditions as those in Fig. 7; however, fluorescence intensity was markedly stronger. This result is consistent with the high RNA concentration observed. Collectively, these results indicate that *Legionella* collaborated with multiple species in the biofilms of non-predatory microorganisms for elongation.

## Discussion

In the environment, *Legionella* species are considered to live parasitically in predatory protozoa (Grimm *et al.*, 1998; Rasch *et al.*, 2016; Caicedo *et al.*, 2018; Dietersdorfer *et al.*, 2018) or symbiotically with non-predatory organisms, such as other bacteria (Taylor *et al.*, 2013), photosynthetic microorganisms (Tison *et al.*, 1980), and algae (Pope *et al.*, 1982). Although open cooling water systems are a typical source of *Legionella* (Fraser *et al.*, 1977; Chamberlain *et al.*, 2017), algae and bacteria are also dominant in cooling towers (Di Gregorio *et al.*, 2017). It currently remains unclear whether non-predatory organisms in these systems contribute to the growth of *Legionella*. Therefore, we collected algae from real cooling water systems and cocultured them with *Legionella* in an artificial oligotrophic medium at an alkaline pH. Quantification and morphological observations of *Legionella* revealed that the algae maintained the *Legionella* 16S rRNA copy number, and also that *Legionella* existed contiguously to the algae, elongating as filamentous cells on algal surfaces. These results are important not only with respect to *Legionella* ecology, but also public health.

### *Legionella* behavior with or without algae under alkaline oligotrophic conditions

Repeated trial-and-error experiments were conducted to optimize culture conditions because there is currently no information on the cultivation of algae sampled from cooling towers in MA medium. Therefore, coculture conditions were not the same, whereas the microorganisms and medium used were the same in all experiments. We considered a comparison of results to help in establishing whether algae affect *Legionella* under alkaline oligotrophic conditions.

The 16S rRNA copy number of *Legionella* cultured with algae (Table 1 No. 1 and 2; Table 2 No. 7 and 10) was repeatedly found to be consistently higher than that of *Legionella* cultured alone (Fig. 1C, 2C, and 6C). On the other hand, in the culture of *Legionella* alone (Table 1 No. 3 and 6; Table 2 No. 8), the 16S rRNA copy number repeatedly decreased by an order of magnitude (Fig. 1C, 2C, and 6C). *Legionella* alone did not appear to be able to maintain its activity under these experimental conditions, which is possible because the *Legionella* culture was in a state of starvation (Li *et al.*, 2015). Moreover, morphological observations using microscopy revealed that *Legionella* cells were present in these samples (Fig. 3D), suggesting that *Legionella* cells maintained their shape even in a state of reduced activity.

However, the CFU count showed varying changes regardless of the presence of algae: its maintenance (Fig. 1A), a decrease to an undetectable level (Fig. 2A), or a decrease (Fig. 6A), which was inconsistent. Mendis *et al.* (2015) previously reported that when cultured in a nutrient-poor medium, the ability of *Legionella* to form CFUs rapidly decreased at temperatures higher than 30°C at pH 4, 5, or 6. The present results support these findings. We attributed the inconsistency in CFUs to different incubation temperatures because higher temperatures were associated with greater reductions in CFUs. Our results also showed that this phenomenon occurred under alkaline conditions. CFUs were maintained under alkaline oligotrophic conditions at 24–34°C for approximately 1 month, mimicking cooling water environments. This is the first study to demonstrate this in a sterile medium, highlighting the necessity of *Legionella* suppression treatments.

As described above, CFUs decreased below the lower detection limit at 36°C; however, the *Legionella* 16S rRNA gene copy number remained unchanged (Fig. 2) and the cell morphology of *Legionella* was preserved (Fig. 3D). Some of the remaining *Legionella* may have been viable but nonculturable (VBNC) because previous studies demonstrated that VBNC *Legionella* formation was induced under oligotrophic conditions (Hwang *et al.*, 2006; Dietersdorfer *et al.*, 2018). These findings indicate that some VBNC *Legionella* strains were resuscitated in cocultures with amoebae. Therefore, it is important to establish a consensus on how to evaluate *Legionella* DNA or VBNC *Legionella* for *Legionella* control in cooling towers.

Although algae and *Legionella* are generally considered to coexist in a symbiotic relationship, only one study reported *Legionella* multiplication in algal biofilms (Pope *et‍ ‍al.*, 1982), and the relationship between algae and *Legionella* in actual cooling towers has yet to be exami­ned. This is the first study in which algae isolated from a real cooling tower were shown to contribute to the survival or growth of *Legionella.* Furthermore, isolated algae, which were dominant in each cooling tower, both promoted *Legionella* survival, and these strains had not previously been reported to coexist with *Legionella*. Therefore, algae that foster *Legionella* may be widespread in cooling water systems. However, the relationship between algae and *Legionella* is complex and has yet to be clarified. Bathia *et al.* (2022) reported that *Legionella* living in hydras were suppressed by Chlorella. Further studies are warranted to elucidate the relationship between algae and *Legionella* because this information is important for a more detailed ecological understanding and the better control of *Legionella*.

On the other hand, the coexistence of *Legionella* with non-predatory organisms other than algae and the pro­liferation of *Legionella* in these settings has been reported,‍ ‍including the formation of *Legionella* CFU under alkaline culture conditions. For example, among photosyn­thetic microorganisms, *Legionella* numbers were shown to increase in cultures with MD medium (pH 7–7.6) in which cyanobacteria proliferated (Tison *et al.*, 1980). Tison *et al.* (1980) showed that *Legionella* numbers decreased at pH >7.5; however, they also detected *Legionella* CFUs at pH ≥8 (data not shown). Other studies revealed that the growth of *Legionella* was promoted by other organisms and that *Legionella* CFUs were detected even under alkaline conditions. Additionally, we focused on oligotrophic conditions at pH 8–8.6 and found that the 16S rRNA copy number of *Legionella* remained high if the algae consortia or pure algae grew. Since alkaline oligotrophic conditions are a common feature of cooling water, this is an important experimental result indicating the ability of *Legionella* to maintain a high activity level in the presence of algae in real cooling systems. Algae always coexist with bacteria in the environment. When *Serratia* isolated from a cooling tower was experimentally added to the coculture of algae and *Legionella*, a further increase was observed in the 16S rRNA copy number (Fig. 6C). Morphological observations revealed strong green fluorescence, which was considered to be derived from *Legionella* (Fig. 8D). Therefore, *Legionella* appear to coexist with two or more species and exhibit better growth under these conditions. This is the main result of this study and warrants further investigation.

### Filamentous morphology of *Legionella* in the coculture with algae

The 16S rRNA copy number of *Legionella* cultured with algae was higher than that of *Legionella* cultured alone. Filamentous cells considered to be *Legionella* were detected in these samples (Fig. 3A, S4, 4, 5B, 7A, and 8D). Even in the coculture with pure algae (Table 2 No. 7), this phenomenon was observed (Fig. 7A and B).

This is the first study in which *in situ* HCR was used to examine *Legionella* in an algal consortium. This fluorescence amplification method is crucial for observing the morphology of *Legionella* in the environment. The only limitations of *in situ* HCR are the non-specific adsorption of amplification probes and the need for the optimization of hybridization and washing conditions for each observation target.

Through the application of *in situ* HCR, *Legionella* was successfully visualized despite autofluorescence by algae, and filamentous extensions were detected on algae. To the best of our knowledge, this is the first study to report these results. In observations of *Legionella* cocultured with the *K. elegans* consortium (Table 1 Culture No. 2), a strong fluorescence signal was observed at both ends of the filament (Fig. 5), which suggested that *Legionella* became filamentous due to growth at both ends without division. However, it remains unclear why *Legionella* elongated without dividing. This coculture incubation was extended to day 54, at which point the *Legionella* CFU count decreased (Fig. 1A), whereas the copy numbers of the 16S rRNA gene (Fig. 1B) and 16S rRNA (Fig. 1C) did not. This contradictory behavior may be attributed to the phenomenon of *Legionella* elongation.

In the present study, *Legionella* on the surface of the algae had rod-shaped or filament-like forms. This morphology differed from that described in other studies. For example, previous studies on *Legionella* growth in the environment were conducted mainly with the coexistence of predatory organisms, such as amoebae and Tetrahymena (Rowbotham, 1980; Grimm *et al.*, 1998; Caicedo *et al.*, 2018). In these cases, *Legionella* appeared as a rod-shaped bacterium randomly within or around the predator cell. Additionally, FISH did not allow us to observe the *Legionella* cell morphology in an artificial biofilm composed of bacteria and protozoa (Taylor *et al.*, 2013). *Legionella* was cocultured with photosynthetic microorganisms, and the biofilm was observed using optical microscopy. In this case, *Legionella* cells were scattered as rod-shaped bacteria in the slime around cyanobacteria (Bohach and Snyder, 1983b).

On the other hand, the filamentation of *Legionella* is a well-known phenomenon. Filamentous *Legionella* species are often identified in environmental water samples or clinical specimens (Blackmon *et al.*, 1978). Paszko-Kolva *et al.* (1992) reported that *Legionella* survived for 1 year in a culture of river water from which microorganisms were removed by filtration, and the morphology of *Legionella* changed to a filamentous nature after approximately 1 year. The present results indicated that algae promoted the elongation of *Legionella*. Paszko-Kolva *et al.* (1992) also stated that morphological changes in *Legionella* were reversible; when filamentous *Legionella* cells were fragmented, they transformed into rods, and rod-shaped *Legionella* cells also transformed into filamentous cells.

Prashar *et al.* (2012, 2018) investigated the mechanisms underlying the infection of pulmonary epithelial cells by filamentous *Legionella* and showed that these cells actively attached to the epithelial cell membrane. Moreover, filamentous *Legionella* attached to and infiltrated cells and then divided into rods in infected cells, in which they proliferated. In the present study, *Legionella* CFUs did not increase; however, the possibility that filamentous *Legionella* infiltrate and proliferate within algae cannot be denied. Therefore, further research will be important for clarifying the mechanisms by which *Legionella* coexist with non-predatory microorganisms in the environment.

In the present study, *Legionella* cells existing closely on algae were rod-shaped or filamentous. This may be related to the culture temperature used in our study (26–28°C). The optimal culture temperature for *Legionella* is 36–37°C, at which *Legionella* become filamentous. To obtain rod-shaped *Legionella*, it is necessary to maintain the culture at 25–30°C. Piao *et al.* (2006) reported that biofilm formation by *Legionella* alone was affected by temperature. An incubation at 36°C produced a filamentous *Legionella* biofilm, while that at 30°C resulted in a biofilm of rod-shaped *Legionella*. Therefore, the impact of temperature on the formation of *Legionella* filaments warrants further research.

**Fig. 9.**
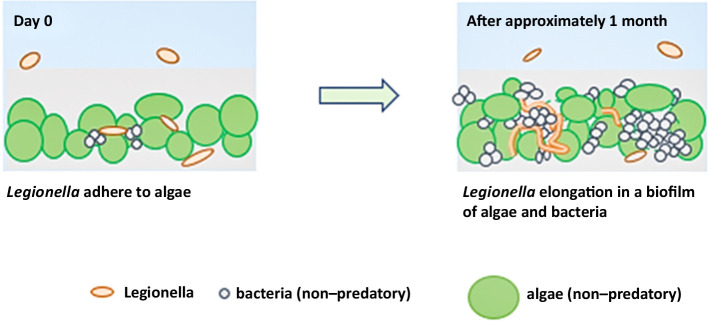
A proposed model of *Legionella* survival and elongation. Photosynthetic microorganisms, such as algae, serve as nutrient sources for bacteria. In addition, *Legionella* species coexist and develop.

We propose a model for *Legionella* survival and growth based on the present results (Fig. 9). Even in alkaline oligotrophic water, *Legionella* elongate when coexisting with living algae, thereby expanding its area. The mechanisms underlying elongation and whether it occurs in cooling towers or the natural environment warrant future investigation.

## Conclusions

We investigated the effects of photosynthetic algae on *Legionella* survival in laboratory experiments. The pres­ent‍ ‍results demonstrated that algae collected from cooling water systems promoted the viability and elongation of *Legionella*, and also that this behavior differed from that of *Legionella* proliferation with predatory organisms, such as amoebae. The main results obtained herein are described below.

1) Although *Legionella* persisted under oligotrophic and alkaline conditions, the *Legionella* 16S rRNA copy number remained high due to its coexistence with live algae. *Legionella* cells that coexisted with live algae elongated at both ends and became filamentous. This morphology of *Legionella* on algal cells was observed for the first time using *in situ* HCR.

2) The two species of algae that were isolated from real cooling towers promoted the viability and elongation of *Legionella*, which suggests that diverse types of algae support *Legionella* in cooling systems.

3) The increase observed in the *Legionella* 16S rRNA copy number was greater in the presence of both algae and bacteria than in the presence of algae alone, indicating that *Legionella* species interact with multiple organisms to improve their survival.

In the present study, non-predatory organisms that are dominant in biofilms promoted *Legionella* elongation under alkaline and oligotrophic conditions. Since *Legionella* species coexist with diverse types of eukaryotic and prokaryotic cells, each symbiotic mechanism needs to be clarified. If these mechanisms are elucidated, it may become possible to control *Legionella* in artificial environments more safely and efficiently.

## Citation

Satou, W., Nagai, N., and Hatamoto, M. (2025) *Legionella* Survives and Elongates in Algal Consortia Containing Bacteria in Alkaline Oligotrophic Conditions. *Microbes Environ ***40**: ME25016.

https://doi.org/10.1264/jsme2.ME25016

## Supplementary Material

Supplementary Material

## Figures and Tables

**Table 1.** Culture conditions and results for *Legionella pneumophila* cultivated with algae consortia.

Culture No.	Alga	Cultivation conditions				Chlorophyll concentration		Appearance of culture
		Temperature (°C)	Light source	Photon flux density (μmol m^–2^ s^–1^)		Initial (μg mg^–1^)	After cultivation (μg mg^–1^)	
1	*V. stellata* consortia	26–28 (room temp.)	Sunlight from North window	8		<Q.D.L.	1.13	green suspension
2	*K. elegans* consortia	26–28 (room temp.)	Sunlight from North window	8		<Q.D.L.	0.92	green suspension
3	—	26 (room temp.)	LED	2		<Q.D.L.	<Q.D.L.	transparent
4	*V. stellata* consortia	36	Fluorescent lamp	105–110		<Q.D.L.	<Q.D.L.	whitening of the alga
5	*K. elegans* consortia	36	Fluorescent lamp	105–110		<Q.D.L.	<Q.D.L.	whitening of the alga
6	—	36	Fluorescent lamp	105–110		<Q.D.L.	<Q.D.L.	transparent

* Quantitative detection limit (Q.D.L.)

**Table 2.** Cultivation conditions and results for *Legionella pneumophila* with pure cultures of *Vischeria stellata* NIES2148 and *Serratia marcescens*.

Culture No.	Bacterium	Alga	Cultivation conditions			Chlorophyll concentration		Appearance of culture
			Temperature (°C)	Light source		Initial (μg mg^–1^)	After cultivation (μg mg^–1^)	
7	*L. pneumophila*	*V. stellata* NIES2148	34	Fluorescent lamp		<Q.D.L.*	226.8	green suspension
8	*L. pneumophila*	—	34	Fluorescent lamp		<Q.D.L.	<Q.D.L.	transparent
9	—	*V. stellata* NIES2148	34	Fluorescent lamp		<Q.D.L.	366.3	green suspension
10	*L. pneumophila* +*S. marcescens*	*V. stellata* NIES2148	34	Fluorescent lamp		<Q.D.L.	238.6	green suspension
11	*L. pneumophila* +*S. marcescens*	—	34	Fluorescent lamp		<Q.D.L.	<Q.D.L.	transparent

* Quantitative detection limit (Q.D.L.)
