# Supplementary material for: Legionella Survives and Elongates in Algal Consortia Containing Bacteria in Alkaline Oligotrophic Conditions
Source: Microbes Environ. 2025 Dec 3;40(4):ME25016. doi: 10.1264/jsme2.ME25016 (PMC12727200; doi:10.1264/jsme2.ME25016)
Supplement: Supplementary file 1 — Supplementary Material [file 40_25016_s1.pdf]

Fig. S1 The Biofilm containing *Vischeria stellata* consortium was gently scraped from the wall of the cooling tower pit or filler using a syringe, a method chosen to collect thin biofilm easily and minimize contamination. Microscopic observation revealed the dominance of a particular species of round alga, identified as *Vischeria stellata*.

Fig. S2 The Biofilm containing *Klebsormidium elegans* consortium was gently scraped from the wall of the cooling tower pit or filler using a syringe, a method chosen to collect thin biofilm easily and minimize contamination. Microscopic observation revealed the dominance of a particular species of oval-shaped colonized alga, identified as *Klebsormidium elegans*.

Fig. S3 FISH detection of *Legionella pneumophila* by an Alexa488-labeled LEG705 probe (green fluorescent) (left) and DAPI staining (right) in co-cultivation of *L. pneumophila* with *Vischeria stellata* consortium on day 27 (condition No. 2 in Table 2). The arrows indicate rod-shaped *L. pneumophila* cells, whose weak fluorescence made it difficult to clearly distinguish their morphology. This was due to the probe's fluorescence being weaker than the autofluorescence emitted by the algae, which made it challenging to distinguish the morphology of *L. pneumophila* cells under the

microscope. Scale bars show 20 μm.

Fig. S4 *in situ* HCR (Hybridization Chain Reaction) observation of *Legionella* morphology co-cultured with *Vischeria stellata* consortium on day 34. *in situ* HCR is a method used to amplify fluorescence signals for visualizing specific bacteria. The LEG705 initiator probe targets *Legionella* DNA, while Alexa488-labeled H1 and H2 amplifier probes enhance the green fluorescence signal. The observed green fluorescence indicates the presence of *Legionella*, while the orange signal represents the autofluorescence of *V. stellata*. Scale bars show 10μm.

Fig. S5 *in situ* HCR observation of each bacterium and alga used in the tri-culture experiment. (A) *Vischeria stellata* NIES2148 showed orange autofluorescence but exhibited no green fluorescence. (B) *Legionella pneumophila* cells that reacted with LEG705 exhibited green fluorescence, but only about 10% as many cells were fluorescent compared to those stained with DAPI. (C) DAPI staining image of *L. pneumophila*, rod-shape cells. (D) *Serratia marcescens* (isolated from cooling tower isolate) produced no green fluorescence. (E) DAPI staining image of *S. marcescens*, which exhibited a short rod-shaped morphology. Scale bar, 20 μm.

Table S1. *in situ* HCR probes for *Legionella* observations

Probe name	Sequence (5'-3') *
LEG705-initiatorH	CCG AAT ACA AAG CAT CAA CGA CTA GAA AAA A -ctg gtg ttc ctt ccg atc
EUB338-initiatorH	CCG AAT ACA AAG CAT CAA CGA CTA GAA AAA A -gct gcc tcc cgt agg agt
NonEUB338-initiatorH	CCG AAT ACA AAG CAT CAA CGA CTA GAA AAA A -act cct acg gga ggc agc
H1-3' Alexa488 label	TCTAGTCGTTGATGCTTTGTATTCGGCGACAGATA -ACCGAATACAAAGCATC
H2-5' Alexa488 label	CCGAATACAAAGCATCAACGACTAGAGATGCTTTG -TATTCGGTTATCTGTCG

* Capital letters indicate the HCR linker and adapter. Lower-case letters correspond to the probe sequence.

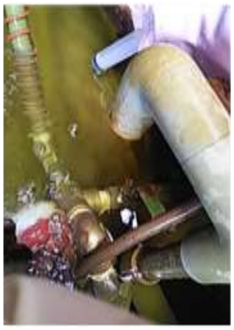

Cooling tower pit

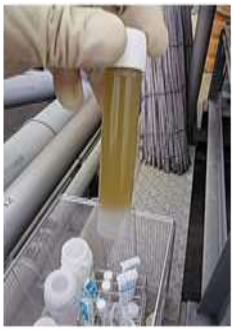

Consortium suspension

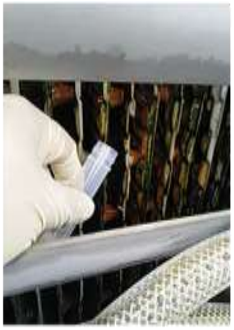

Cooling tower filler

Fig. *Vischeria stellata* consortium sampling points

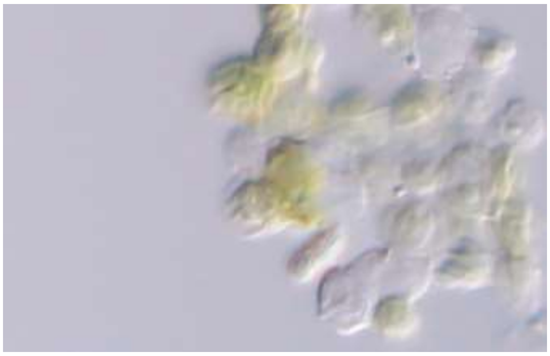

Microscopic observation of biofilm contained *Vischeria stellata* consortium

Fig.S1 wakako satou

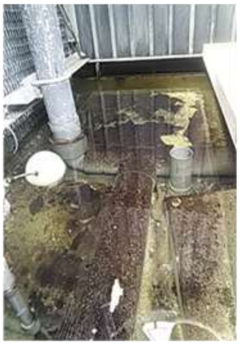

Cooling tower pit

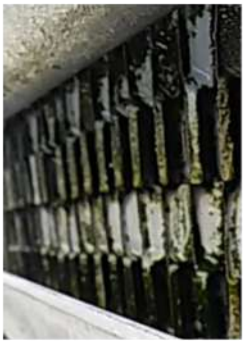

Cooling tower filler

Fig. *Klebsormidium elegans* consortium sampling points

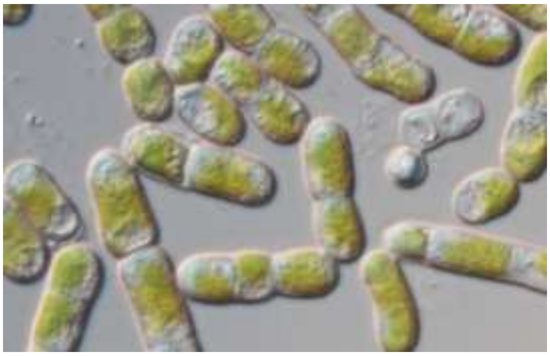

Microscopic observation of biofilm contained
Klebsormidium elegans consortium

FigS2 wakako satou

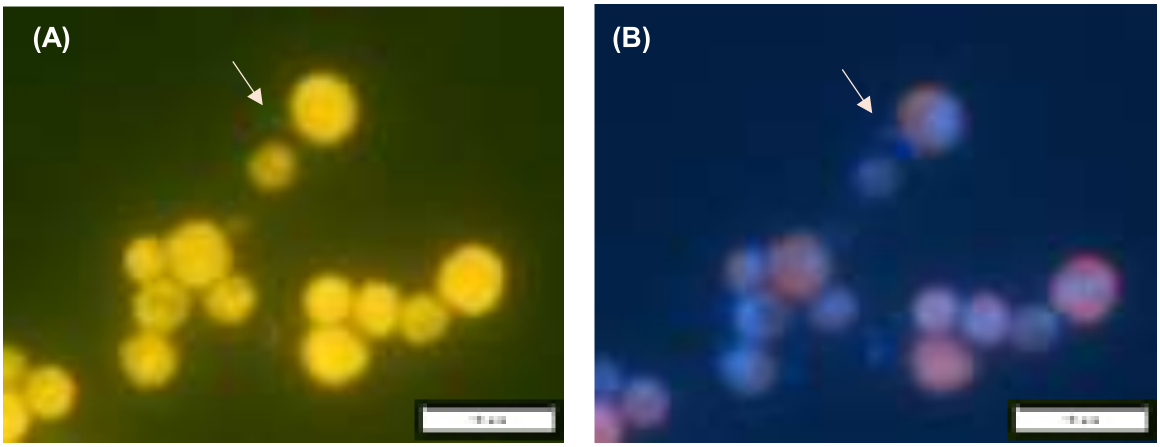

Fig.S3 wakako satou

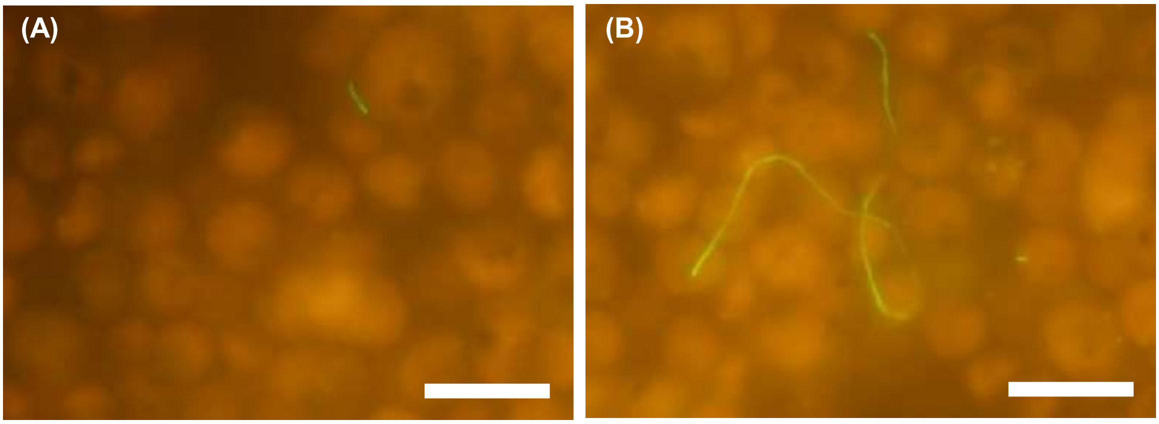

Fig.S4 wakako satou

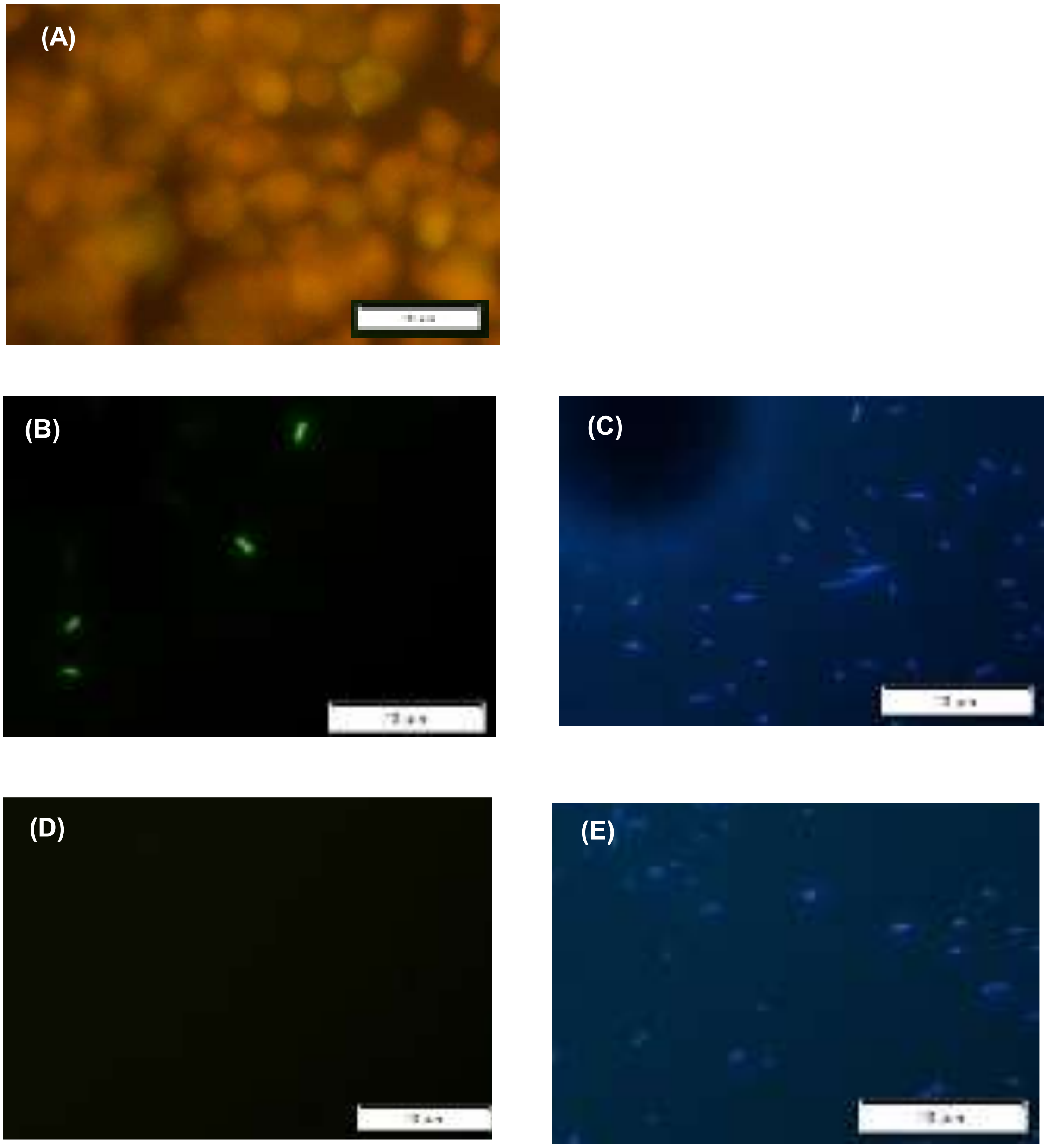

Fig.S5 wakako satou
